# Supplementary material for: Transcriptional repression facilitates RNA:DNA hybrid accumulation at DNA double-strand breaks
Source: Nat Cell Biol. 2025 May 30;27(6):992–1005. doi: 10.1038/s41556-025-01669-y (PMC12173947; doi:10.1038/s41556-025-01669-y)
Supplement: Supplementary file 1 — Reporting Summary [file 41556_2025_1669_MOESM1_ESM.pdf]

nature portfolio

Corresponding author(s): Gaelle Legube, Aline Marnef, Thomas Clouaire

Last updated by author(s): Mar 20, 2025

# Reporting Summary

Nature Portfolio wishes to improve the reproducibility of the work that we publish. This form provides structure for consistency and transparency in reporting. For further information on Nature Portfolio policies, see our Editorial Policies and the Editorial Policy Checklist.

## Statistics

For all statistical analyses, confirm that the following items are present in the figure legend, table legend, main text, or Methods section.

| n/a | Confirmed | |
|---|---|---|
| ☐ | ☒ | The exact sample size (*n*) for each experimental group/condition, given as a discrete number and unit of measurement |
| ☐ | ☒ | A statement on whether measurements were taken from distinct samples or whether the same sample was measured repeatedly |
| ☐ | ☒ | The statistical test(s) used AND whether they are one- or two-sided<br>*Only common tests should be described solely by name; describe more complex techniques in the Methods section.* |
| ☒ | ☐ | A description of all covariates tested |
| ☒ | ☐ | A description of any assumptions or corrections, such as tests of normality and adjustment for multiple comparisons |
| ☐ | ☒ | A full description of the statistical parameters including central tendency (e.g. means) or other basic estimates (e.g. regression coefficient) AND variation (e.g. standard deviation) or associated estimates of uncertainty (e.g. confidence intervals) |
| ☒ | ☐ | For null hypothesis testing, the test statistic (e.g. *F*, *t*, *r*) with confidence intervals, effect sizes, degrees of freedom and *P* value noted<br>*Give P values as exact values whenever suitable.* |
| ☒ | ☐ | For Bayesian analysis, information on the choice of priors and Markov chain Monte Carlo settings |
| ☒ | ☐ | For hierarchical and complex designs, identification of the appropriate level for tests and full reporting of outcomes |
| ☒ | ☐ | Estimates of effect sizes (e.g. Cohen's *d*, Pearson's *r*), indicating how they were calculated |

*Our web collection on statistics for biologists contains articles on many of the points above.*

## Software and code

Policy information about availability of computer code

| | |
|---|---|
| Data collection | Bio-Rad CFX Manager version 3.1<br>ChemiDoc™ Touch Imaging System.and Image Lab Touch version 5.2.1<br>MetaMorph version 7.1.0.0 |
| Data analysis | Cell Profiler 4.2.8<br>Icy software version 2.0.2.0 (https://icy.bioimageanalysis.org/ software )<br>Bio-Rad CFX Manager version 3.1<br>QuantaSoft version 1.74.4 (Bio-Rad)<br>bwa (https://bio-bwa.sourceforge.net/) version 0.7.12-r1039<br>samtools (http://www.htslib.org/) version 1.9<br>bamCoverage from deepTools (https://deeptools.readthedocs.io/en/develop/)<br>R 4.2<br>GenomicRanges 1.38<br>plyranges 1.6.10<br>Integrated Genome Browser version 9.1.6 (https://www.bioviz.org)<br>Integrated Genome Viewer version 2.19.1 (https://igv.org)<br>deeptools 3.4.3<br>rtracklayer 1.58.0<br>stringr 1.5.0<br>dplyr 1.0.10 |

ggplot2 3.4.0
rstatix 0.7.1

For manuscripts utilizing custom algorithms or software that are central to the research but not yet described in published literature, software must be made available to editors and reviewers. We strongly encourage code deposition in a community repository (e.g. GitHub). See the Nature Portfolio guidelines for submitting code & software for further information.

## Data

Policy information about availability of data

All manuscripts must include a data availability statement. This statement should provide the following information, where applicable:

- Accession codes, unique identifiers, or web links for publicly available datasets
- A description of any restrictions on data availability
- For clinical datasets or third party data, please ensure that the statement adheres to our policy

All high-throughput sequencing data (ChIP-seq, qDRIP-seq, END-seq, TTchem-seq, DRB/TTchem-seq and RNA-seq) have been deposited to Array Express (https://www.ebi.ac.uk/arrayexpress/) under accession number E-MTAB-13197.
The hg19 reference genome was obtained from UCSC (https://hgdownload.soe.ucsc.edu/goldenPath/hg19/bigZips/).

## Human research participants

Policy information about studies involving human research participants and Sex and Gender in Research.

| | |
|---|---|
| Reporting on sex and gender | Not relevant to our study. |
| Population characteristics | NA |
| Recruitment | NA |
| Ethics oversight | NA |

Note that full information on the approval of the study protocol must also be provided in the manuscript.

# Field-specific reporting

Please select the one below that is the best fit for your research. If you are not sure, read the appropriate sections before making your selection.

☒ Life sciences ☐ Behavioural & social sciences ☐ Ecological, evolutionary & environmental sciences

For a reference copy of the document with all sections, see nature.com/documents/nr-reporting-summary-flat.pdf

# Life sciences study design

All studies must disclose on these points even when the disclosure is negative.

**Sample size**

No sample size calculation was performed. The number of samples in each experiment was determined based on standards practice in the field (see reference 35, 39 and 43 for exemples) in most cases in triplicates or above. The number of independent experiments are indicated in the legend of each Figure.

**Data exclusions**

No data were excluded from analysis.

**Replication**

- ChIP-seq before and after DSB induction: n=1. However, sequenced DNA comes from a pool of independent ChIP: n=10 for SPIN1, n=12 for PAF1, n=1 for SPT5, n=4 for RNAPII total (405), n=3 for RNAPII total (D8L4Y); n=6 for Y1P, n=4 for S2P (3E10), n=3 for S2P (E1Z3G), n=4 for S5P (3E8), n=3 for S5P (D9NI), n=4 for S7P, n=5 for POLR3A, n=5 for POLR3E). ChIP-seq before and after 4h and 24h DSB induction: n=1 (but sequenced DNA from a pool of independent ChIP: n=9 RPA)
- qDRIP-seq before and after 4h and 24h DSB induction: n=1 (but sequenced DNA from a pool of 3 independent qDRIP). qDRIP-seq before and after DSB induction in siCtrl and siSPIN1 transfected cells: n=1 (but sequenced DNA from a pool of 3 independent qDRIP).
-TTchem-seq or DRB/TTchem-seq: n=1
- DRIP-qPCR: n≥3
- END-seq before and 4h and 24h after DSB induction: n=1. END-seq before and after DSB induction in siRNA transfected cells: n=1
- RT-qPCR in siRNA transfected cells and MS31-treated cells: n≥3
- Resection assay: n≥3
- All ChIP-qPCR n≥3 apart from that after DSB induction with MS31-treated cells: n=1.
- Clonogenic assay before and after DSB induction with siRNA transfected cells: n≥3. Clonogenic assay before and after DSB induction with MS31-treated and MS31-/Etoposide-treated cells and Mirin-treated cells: n≥3.
- Western blots: representative experiments are shown for siRNA-mediated depletion efficiency out of n=2 for CtIP, n=3 for SETX, n=2 for NELF-E, n=4 for SPIN1. For siRNA-mediated depletion efficiency of PAF1 and SKI8 and for SPIN1 and H3K4me3 levels upon MS31 treatment n=1.

- Fluorescence microscopy: n=1 analysis on more than 100 cells per conditions
- Illegitimate re-joining: frequency n=4.

Randomization

Randomization is not relevant because we did not use different experimental groups in our study.

Blinding

Blinding was not relevant to our study since we did not have experimental group to compare.

# Reporting for specific materials, systems and methods

We require information from authors about some types of materials, experimental systems and methods used in many studies. Here, indicate whether each material, system or method listed is relevant to your study. If you are not sure if a list item applies to your research, read the appropriate section before selecting a response.

## Materials & experimental systems

| n/a | Involved in the study |
|---|---|
| ☐ | ☒ Antibodies |
| ☐ | ☒ Eukaryotic cell lines |
| ☒ | ☐ Palaeontology and archaeology |
| ☒ | ☐ Animals and other organisms |
| ☒ | ☐ Clinical data |
| ☒ | ☐ Dual use research of concern |

## Methods

| n/a | Involved in the study |
|---|---|
| ☐ | ☒ ChIP-seq |
| ☒ | ☐ Flow cytometry |
| ☒ | ☐ MRI-based neuroimaging |

# Antibodies

Antibodies used

RNAPII (405A) Bethyl A304-405A 2 µL/200 µg of DNA ChIP
RNAPII (D8L4Y) Cell Signaling 14958 7 µL/ 100 µg of DNA ChIP
POLR3A Abcam ab96328 6 µL/150 µg of DNA ChIP
POLR3E Bethyl A303-707A 5 µL/150 µg of DNA ChIP
Y1P Active Motif 61383 3 µL/200 µg of DNA ChIP
S2P (3E10) Chromotek 3E10 75 µL/150 µg of DNA ChIP
S2P (E1Z3G) Cell Signaling 13499 7 µL/ 100 µg of DNA ChIP
S5P (3E8) Chromotek 3E8 75 µL/150 µg of DNA ChIP
S5P (D9N5I) Cell Signaling 13523 7 µL/ 100 µg of DNA ChIP
S7P Chromotek 4E12 75 µL/150 µg of DNA ChIP
S9.6 Antibodies Incorporated 6 µg/4 µg of DNA DRIP
SPT5 Bethyl A3030-707A 2.5 µL/100 µg of DNA ChIP
PAF1 Abcam ab137519 2 µL/150 µg of DNA ChIP
1:1000 WB
SKI8 (WDR61) Invitrogen PA540079 1:500 WB
RPA Abcam ab10359 4 µg/200 µg ChIP
gH2AX Abcam ab176458 1 µL/200 µg of DNA ChIP
RAD51 Abcam ab176458 2 µL/200 µg of DNA ChIP
RAD51 sc8349 1:100 IF
H3K4me3 Abcam ab8580 1 µL/25 µg of DNA ChIP
1:1000 WB
SPIN1 Proteintech, 12105-1-AP 7 µL/200 µg of DNA ChIP
1:1000 WB
NELF-E Abcam Ab170104 1:1000 WB
Myosin Sigma, M3567 1:2000 WB
Tubulin Sigma T6199 1:10000 WB
Anti-rabbit-HRP Sigma A0545 1:10000 WB
Anti-mouse-HRP Sigma, A2554 1:10000 WB
gH2AX Millipore JBW301 1:1000 IF
BRCA1 Calbiochem OP92-100UG 1:500 IF

Validation

SPT5 (Bethyl A300-869A) validate in ChIP (https://pubmed.ncbi.nlm.nih.gov/35325203/)
PAF1 (Abcam, ab137519) validated in ChIP (https://pubmed.ncbi.nlm.nih.gov/33852864/) and western blot (https://pubmed.ncbi.nlm.nih.gov/30367041/)
SKI8 (Invitrogen PA540079) validated in western blot (https://www.thermofisher.com/antibody/product/WDR61-Antibody-Polyclonal/PA5-40079)
SPIN1 (Proteintech, 12105-1-AP) validated in ChIP (https://pubmed.ncbi.nlm.nih.gov/36736887/) and in western blot (https://www.ptglab.com/fr/products/SPIN1-Antibody-12105-1-AP.htm)
RNAPII (Bethyl A304-405A) validated in ChIP (https://www.fortislife.com/products/primary-antibodies/rabbit-anti-rna-polymerase-ii-antibody/BETHYL-A304-405)
RNAPII (Cell signalling D8L4Y) validated in Chip (https://pubmed.ncbi.nlm.nih.gov/30472187/)
POLR3A (Abcam ab96328) validated in ChIP (https://pubmed.ncbi.nlm.nih.gov/35637192/)
POLR3E (Bethyl A3030-707A) validated in ChIP with our data
Y1P (Active Motif 61383) validated in ChIP (https://doi.org/10.1016/j.celrep.2016.05.010)

S2P (Chromotek 3E10) validated in ChIP (https://pubmed.ncbi.nlm.nih.gov/24478330/)
S2P (E1Z3G) validated in ChIP (https://pubmed.ncbi.nlm.nih.gov/28782042/)
S5P (Chromotek 3E8) validated in ChIP (https://pubmed.ncbi.nlm.nih.gov/24478330/)
S5P (D9NI) validated in ChIP (https://pubmed.ncbi.nlm.nih.gov/24478330/)
S7P (Chromotek 4E12) validated in ChIP (https://pubmed.ncbi.nlm.nih.gov/24478330/)
S9.6 (Antibodies Incorporated) validated in DRIP (https://academic.oup.com/nar/article/48/14/e84/5858111)
RPA32/2 (Abcam ab10359) validated in ChIP (https://genesdev.cshlp.org/content/35/19-20/1356.long)
NELF-E (Abcam ab170104) validated in Western Blot (https://www.abcam.com/products/primary-antibodies/nelfe-antibody-epr11600-ab170104.html#lb)
Myosin (Sigma, M3567) validated in Western Blot (https://www.sigmaaldrich.com/FR/fr/search/m3567?focus=products&page=1&perpage=30&sort=relevance&term=m3567&type=product)
H3K4me3 (Abcam ab8580) validated in ChIP and Western Blot (abcam.com/products/primary-antibodies/histone-h3-tri-methyl-k4-antibody-chip-grade-ab8580.html)
α-Tubulin (Sigma T6199) validated in Western Blot (https://www.sigmaaldrich.com/FR/fr/product/sigma/t6199)
Anti-rabbit-HRP (Sigma A0545) validated in Western Blot (https://www.sigmaaldrich.com/FR/fr/product/sigma/a0545)
Anti-mouse-HRP (Sigma, A2554) validated in Western Blot (https://www.sigmaaldrich.com/FR/fr/product/sigma/a2554)
gH2AX (Abcam ab81299) validated in ChIP (https://doi.org/10.1016/j.molcel.2018.08.020)
gH2AX (Millipore JBW301) validated in IF (https://www.sigmaaldrich.com/FR/fr/product/mm/05636)
RAD51 (Abcam ab176458) validated in ChIP (https://www.ncbi.nlm.nih.gov/pmc/articles/PMC6993210/)
RAD51 (Santacruz, sc8349) validated in IF (https://pubmed.ncbi.nlm.nih.gov/29416069/)

## Eukaryotic cell lines

Policy information about cell lines and Sex and Gender in Research

| | |
|---|---|
| Cell line source(s) | Cell lines developped from U2OS cells (ATCC® HTB-96™) in Gaelle Legube's laboratory (DIvA cell line and AID-DIvA cell line). Lenti-X HEK293T were purchased from Clontech (632180). HAP1 cells were purchased from Horizon (C859). |
| Authentication | Authentication of the U20S cell line was performed by the provider ATCC which uses morphology, karyotyping and PCR based approaches to confirm the identitity of human cell lines. DIvA and AID-DIvA , both derived from U20S cells, Lenti-X HEK293T and HAP1 cells were not further authenticated. |
| Mycoplasma contamination | All cell lines (DIvA, AID-DIvA, Lenti-X HEK293T and HAP1) were regularly tested for absence of mycoplasma contamination by using the TransDetect® PCR Mycoplasma Detection Kit (TransGen Biotech). All cell lines used in this study were tested negative for Mycoplasma. |
| Commonly misidentified lines (See ICLAC register) | No commonly misidentified cell lines were used in the study. Cell lines used in the study are not registered in ICLAC. |

## Methodology

### Replicates

- ChIP-seq before and after DSB induction: n=1. However, sequenced DNA comes from a pool of independent ChIP: n=10 for SPIN1, n=12 for PAF1, n=1 for SPT5, n=4 for RNAPII total (405), n=3 for RNAPII total (D8L4Y); n=6 for Y1P, n=4 for S2P (3E10), n=3 for S2P (E1Z3G), n=4 for S5P (3E8), n=3 for S5P (D9NI), n=4 for S7P, n=5 for POLR3A, n=5 for POLR3E). ChIP-seq before and after 4h and 24h DSB induction: n=1 (but sequenced DNA from a pool of independent ChIP: n=9 RPA)
- qDRIP-seq before and after 4h and 24h DSB induction: n=1 (but sequenced DNA from a pool of 3 independent qDRIP). qDRIP-seq before and after DSB induction in siCtrl and siSPIN1 transfected cells: n=1 (but sequenced DNA from a pool of 3 independent qDRIP).
-TTchem-seq or DRB/TTchem-seq: n=1

### Sequencing depth

RNAPOLII_Total_405_mOHT - 138 million single end reads (85nt)
RNAPOLII_Total_405_pOHT - 152 million single end reads (85nt)
RNAPOLII_Total_D8L4Y_mOHT - 48 million single end reads (122nt)
RNAPOLII_Total_D8L4Y_pOHT - 27 million single end reads (122nt)
POLR3A_mOHT - 17 million single end reads (85nt)
POLR3A_pOHT - 12.8 million single end reads (85nt)
POLR3E_mOHT - 22.3 million single end reads (85nt)
POLR3E_pOHT - 11.6 million single end reads (85nt)
RNAPOLII_Y1P_mOHT - 96.6 million single end reads (85nt)
RNAPOLII_Y1P_pOHT - 86.5 million single end reads (85nt)
RNAPOLII_S2_3E10_mOHT - 107 million single end reads (85nt)
RNAPOLII_S2_3E10_pOHT - 115 million single end reads (85nt)
RNAPOLII_S2_E1Z3G_mOHT - 84 million single end reads (122nt)
RNAPOLII_S2_E1Z3G_pOHT – 69 million single end reads (122nt)
RNAPOLII_S5_3E8_mOHT - 148 million single end reads (85nt)
RNAPOLII_S5_3E8_pOHT - 133 million single end reads (85nt)
RNAPOLII_S5_D9NI_mOHT - 65 million single end reads (122nt)
RNAPOLII_S5_D9NI_pOHT - 86 million single end reads (122nt)
RNAPOLII_S7_mOHT - 126 million single end reads (85nt)
RNAPOLII_S7_pOHT - 152 million single end reads (85nt)
Spin1_mOHT - 23.2 million paired end reads (80nt)
Spin1_pOHT - 31.3 million paired end reads (80nt)
PAF1_DIVA - 49 million single end reads (122 nt)
PAF1_OHT - 38 million single end reads (122 nt)
SPT5_DIVA - 129 million single end reads (132 nt)
SPT5_OHT - 143 million single end reads (132 nt)
RPA_mOHT - 86.2 million paired end reads (75nt)
RPA_pOHT_4H - 77.2 million paired end reads (75nt)
RPA_pOHT_24H - 70.2 million paired end reads (75nt)
qDRIP_DIVA - 83 million paired end reads (75nt)
qDRIP_OHT - 82 million paired end reads (75nt)
qDRIP_OHT_24h - 92 million paired end reads (75nt)
ENDseq_NT – 29 million reads paired end reads (61nt)
ENDseq_Etoposide_rep1 - 39 million reads paired end reads (61nt)
ENDseq_Etoposide_rep2 -56 million reads paired end reads (61nt)
ENDseq_siCTRL_DIVA - 65 million reads paired end reads (61nt)
ENDseq_siCTRL_OHT - 65 million reads paired end reads (61nt)
ENDseq_siSETX_DIVA -71 million reads paired end reads (61nt)
ENDseq_siSETX_OHT -71 million reads paired end reads (61nt)
TTchemseq_no4SU – 53 million reads single end reads (75nt)
TTchemseq_DIVA– 64 million reads single end reads (75nt)
TTchemseq_OHT – 66 million reads single end reads (75nt)
DRB_TTchemseq_DIVA_DRB – 62 million reads single end reads (75nt)
DRB_TTchemseq_DIVA _DRB_5min - 70 million reads single end reads (75nt)
DRB_TTchemseq_DIVA _DRB_10min – 75 million reads single end reads (75nt)
DRB_TTchemseq_DIVA _DRB_20min – 97 million reads single end reads (75nt)
DRB_TTchemseq_DIVA _DRB_30min – 73 million reads single end reads (75nt)
DRB_TTchemseq_DIVA _DRB_40min – 107 million reads single end reads (75nt)
DRB_TTchemseq_OHT_DRB — 65 million reads single end reads (75nt)
DRB_TTchemseq_OHT_DRB_5min - 52 million reads single end reads (75nt)
DRB_TTchemseq_OHT _DRB_10min – 61 million reads single end reads (75nt)
DRB_TTchemseq_OHT _DRB_20min – 75 million reads single end reads (75nt)
DRB_TTchemseq_OHT _DRB_30min – 72 million reads single end reads (75nt)
DRB_TTchemseq_DRB_OHT _40min – 59 million reads single end reads (75nt)

### Antibodies

RNAPII (405A) Bethyl A304-405A 2 µL/200 µg of DNA ChIP
RNAPII (D8L4Y) Cell Signaling 14958 7 µL/ 100 µg of DNA ChIP
POLR3A Abcam ab96328 6 µL/150 µg of DNA ChIP
POLR3E Bethyl A303-707A 5 µL/150 µg of DNA ChIP
Y1P Active Motif 61383 3 µL/200 µg of DNA ChIP
S2P (3E10) Chromotek 3E10 75 µL/150 µg of DNA ChIP
S2P (E1Z3G) Cell Signaling 13499 7 µL/ 100 µg of DNA ChIP
S5P (3E8) Chromotek 3E8 75 µL/150 µg of DNA ChIP
S5P (D9N5I) Cell Signaling 13523 7 µL/ 100 µg of DNA ChIP

# ChIP-seq

## Data deposition

☒ Confirm that both raw and final processed data have been deposited in a public database such as GEO.

☐ Confirm that you have deposited or provided access to graph files (e.g. BED files) for the called peaks.

**Data access links**
*May remain private before publication.*

E-MTAB-13197

**Files in database submission**

RNAPOLII_Total_mOHT
RNAPOLII_Total_pOHT
POLR3A_mOHT
POLR3A_pOHT
POLR3E_mOHT
POLR3E_pOHT
RNAPOLII_Y1P_mOHT
RNAPOLII_Y1P_pOHT
RNAPOLII_S2_mOHT
RNAPOLII_S2_pOHT
RNAPOLII_S5_mOHT
RNAPOLII_S5_pOHT
RNAPOLII_S7_mOHT
RNAPOLII_S7_pOHT
Spin1_mOHT
Spin1_pOHT
RPA_mOHT
RPA_pOHT_4H
RPA_pOHT_24H

**Genome browser session**
(e.g. UCSC)

No longer applicable

S7P Chromotek 4E12 75 µL/150 µg of DNA ChIP
S9.6 Antibodies Incorporated 6 µg/4 µg of DNA DRIP
SPT5 Bethyl A3030-707A 2.5 µL/100 µg of DNA ChIP
PAF1 Abcam ab137519 2 µL/150 µg of DNA ChIP
RPA Abcam ab10359 4 µg/200 µg ChIP
gH2AX Abcam ab176458  1 µL/200 µg of DNA ChIP
RAD51 Abcam ab176458 2 µL/200 µg of DNA ChIP
H3K4me3 Abcam ab8580 1 µL/25 µg of DNA ChIP
SPIN1 Proteintech, 12105-1-AP 7 µL/200 µg of DNA ChIP

**Peak calling parameters**

Peak calling was not performed in this study

**Data quality**

All sequencing data has been submitted to fastqc analysis. During the sam to bam step, sequencing reads has been filtered based on their Phred score (-q 25).

**Software**

bedtools v2.26.0
bwa 0.7.12-r1039
samtools 1.9
deeptools 3.4.3
R 4.2
