# Supplementary material for: Transcriptional repression facilitates RNA:DNA hybrid accumulation at DNA double-strand breaks
Source: Nat Cell Biol. 2025 May 30;27(6):992–1005. doi: 10.1038/s41556-025-01669-y (PMC12173947; doi:10.1038/s41556-025-01669-y)

Extended Data Fig. 4i

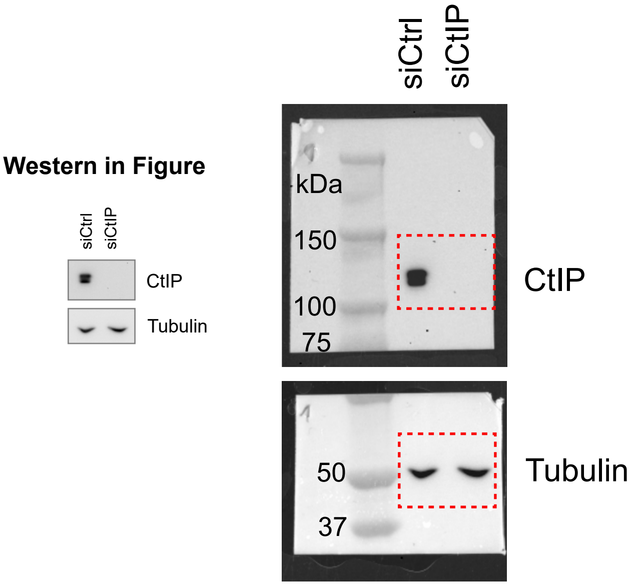

Extended Data Fig. 5d

Western in Figure

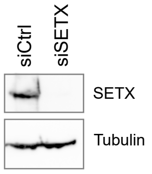

Merge with ladder

long exposure

Short exposure

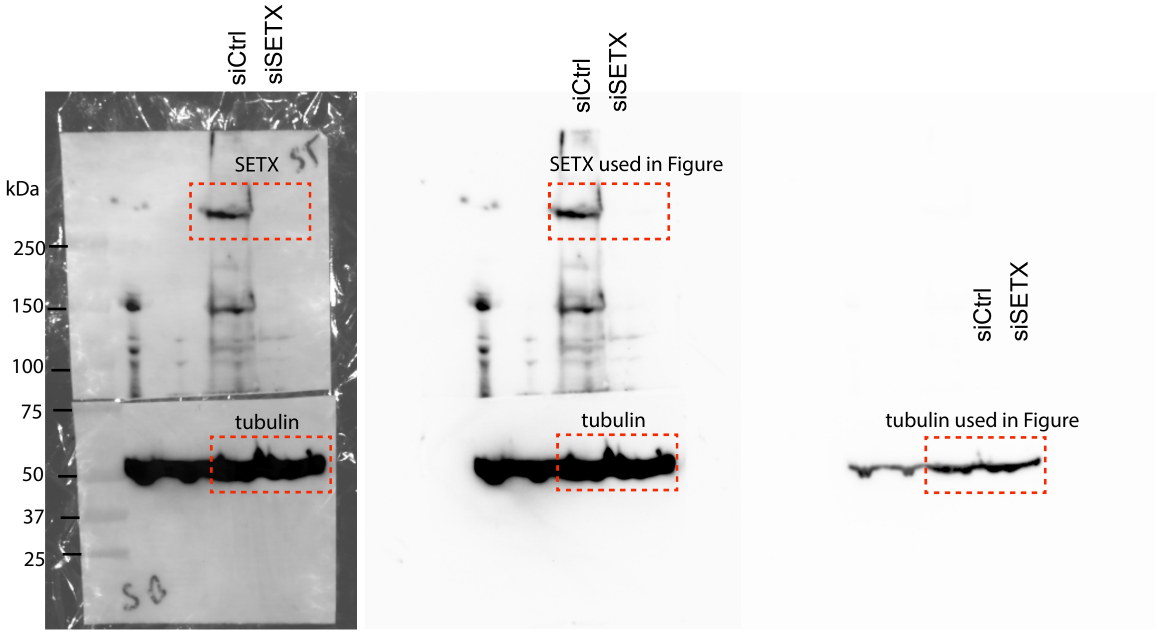

Extended Data Fig. 7b

Western in Figure

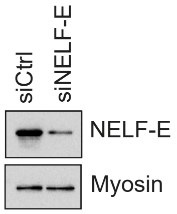

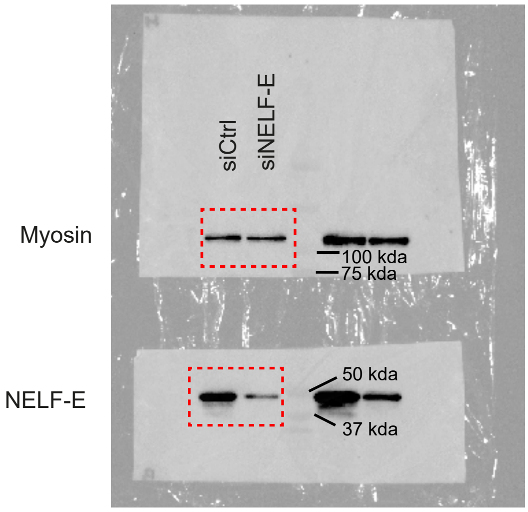

Extended Data Fig. 7g

Westerns in Figure

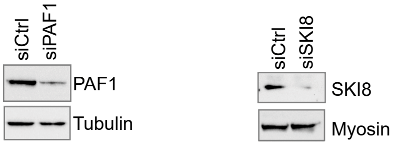

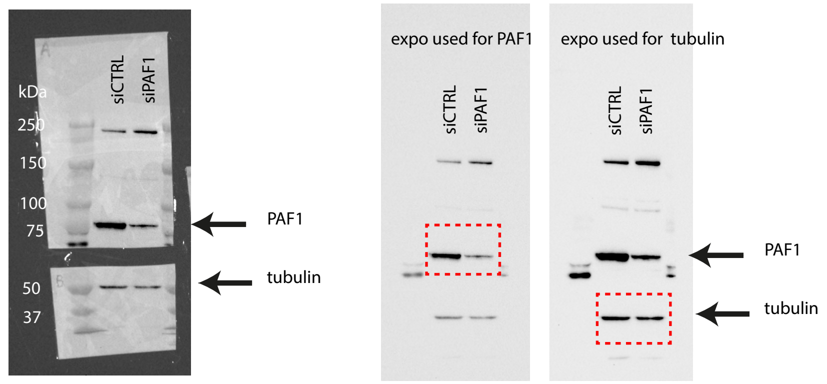

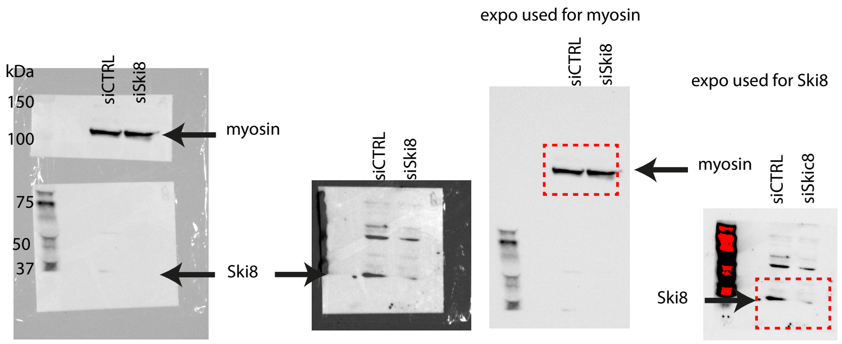

Extended Data Fig. 8d

Western in Figure

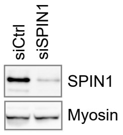

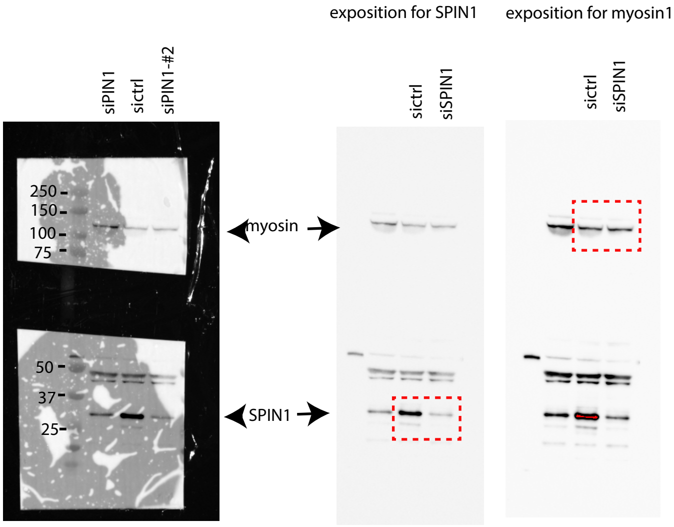

Extended Data Fig. 8e

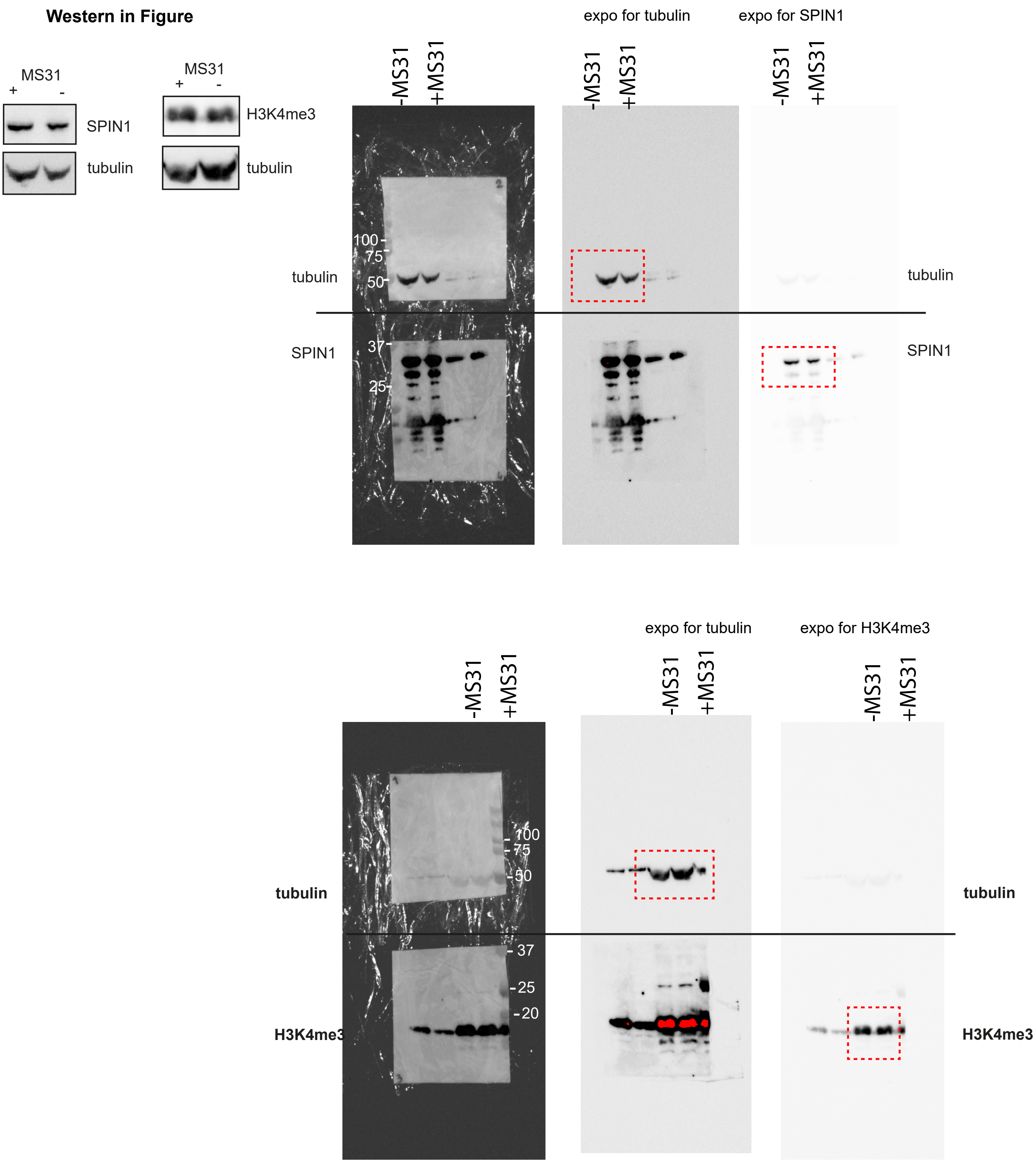

Supplement: Supplementary file 5 — Unprocessed western blots. [file 41556_2025_1669_MOESM5_ESM.pdf]
